# Resistance Training Combined With Cognitive Training Increases Brain Derived Neurotrophic Factor and Improves Cognitive Function in Healthy Older Adults

**DOI:** 10.3389/fpsyg.2022.870561

**Published:** 2022-10-14

**Authors:** Luz Albany Arcila Castaño, Vivian Castillo de Lima, João Francisco Barbieri, Erick Guilherme Peixoto de Lucena, Arthur Fernandes Gáspari, Hidenori Arai, Camila Vieira Ligo Teixeira, Hélio José Coelho-Júnior, Marco Carlos Uchida

**Affiliations:** ^1^Applied Kinesiology Laboratory, Department of Adapted Physical Activity, School of Physical Education, University of Campinas, Campinas, Brazil; ^2^Laboratory of Electromyography Studies, Department of Sport Science, School of Physical Education, University of Campinas, Campinas, Brazil; ^3^National Center for Geriatrics and Gerontology, Obu, Aichi, Japan; ^4^NIH Biomedical Research Center, University of Maryland, Baltimore, Baltimore, MD, United States

**Keywords:** BDNF, cognitive training, dual task, resistance training, older adult

## Abstract

**Background:**

The present study compared the effects of a traditional resistance training (TRT) and resistance training combined with cognitive task (RT + CT) on body composition, physical performance, cognitive function, and plasma brain-derived neurotrophic factor (BNDF) levels in older adults.

**Methods:**

Thirty community-dwelling older adults were randomized into TRT (70.0 ± 8.1; 25% men) and RT + CT (66.3 ± 4.6; 31% men). Exercise groups performed a similar resistance training (RT) program, twice a week over 16 weeks. Cognitive Training involved performing verbal fluency simultaneously with RT. Exercise sessions (eight resistance exercises) were performed 2–3 sets, 8–15 repetitions at 60%–70% of 1-repetition maximum (1RM). Body composition, physical function, cognitive performance, and BDNF levels were assessed before and after intervention period.

**Results:**

The physical performance was similarly improved in response to both TRT and RT + CT (*p* = 0.001). However, exclusive improvements on cognitive function (*p* < 0.001) and BDNF levels (*p* = 0.001) were observed only after RT + CT.

**Conclusion:**

The RT program associated with a cognitive task, improved physical and cognitive performance in healthy older adults.

## Introduction

The aging process involves progressive organic deterioration and a significant decline in physiological functions, potentially leading to the development of geriatric syndromes and chronic diseases (Salthouse, 2013; World Health Organization, 2018; Cruz-Jentoft et al., 2019; Hoogendijk et al., 2019). According to the World Health Organization, attention should be paid to the age-related decrease in neuromuscular performance and cognitive functioning, given their close relationship with functional independence and healthy aging (Pruchno and Carr, 2017; World Health Organization, 2018).

Aging affects negatively the neuromuscular function by regulating multiple mechanisms, reducing mobility, muscle mass, strength and power (Cruz-Jentoft et al., 2019). Muscle atrophy and decreased physical performance are diagnostic criteria for frailty (Hoogendijk et al., 2019), sarcopenia (Cruz-Jentoft et al., 2019) and risk factors for adverse health events, including falls (Shumway-Cook et al., 2000; Buatois et al., 2008).

Cognition refers to mental processes responsible for the interaction between body and environment. Cognitive functions, such as memory, attention, and executive functions are affected by aging as well (Salthouse, 2013). Furthermore, age-related decline in cognitive functions affects the ability to perform activities of daily living (Gold, 2012). We can evaluate cognitive function in diverse ways. One of them, is verbal abilities. Verbal abilities involve the capacity to retrieve grammatical representations and word sounds. This function are assessed by verbal fluency (VF) tasks. These abilities are crucial for communication and interaction with the world (Whiteside et al., 2016).

Declines caused by age encompass physical and cognitive aspects, which profoundly affect daily life activities, since this model of activity involves simultaneous motor and cognitive tasks (dual task). Aging impairs this ability to perform dual task as walking and talking at the same time, what is seen as a poor health prognosis (Shumway-Cook et al., 2000; Al-Yahya et al., 2019). One way to improve the ability to performance dual task is to combine physical training with cognitive training (CT) at the same time (Herold et al., 2018). Many mechanisms can mediate the effects of dual task on cognitive performance (Chang et al., 2012). For instance, brain-derived neurotrophic factor (BDNF) is involved in neurogenesis, synaptic plasticity, neuronal morphology, and neuropathology (Sujin et al., 2019). Although a theoretical basis for the exercise-induced improvement in cognitive performance by BDNF has been proposed (Kim et al., 2019), studies have shown that not all kind of physical activity can improve BDNF level, as seen the limited effect of resistance training (RT) on BDNF level (Fragala et al., 2014; Prestes et al., 2015).

The maintenance of neuromuscular and cognitive performance is an important topic in geriatric sciences and many therapies that can potentially accomplish this goal have been proposed in the past few years. For instance, it has been argued that RT should be considered a first-line therapy to counteract age-related neuromuscular deterioration (Chodzko-Zajko et al., 2009; Fragala et al., 2019). These assumptions are based on original studies (Coelho-Júnior et al., 2019) and systematic reviews (Borde et al., 2015), in which RT improved neuromuscular performance in older adults. Although studies have shown contradictory effects of RT on cognitive functions, having either no effects (Tsutsumi et al., 1997; Kimura et al., 2010) or positive effect (Cassilhas et al., 2007; Liu-Ambrose et al., 2010; Coelho-Júnior et al., 2020). To the best of our knowledge, no studies have assessed the impact of RT + CT on neuromuscular and cognitive function in older adults. We hypothesize that traditional RT (TRT) and RT + CT can improve neuromuscular function, but only RT + CT can improve both, neuromuscular and cognitive function.

## MATERIALS AND Methods

### Experimental Design

This two-arm parallel randomized trial compared the effects of TRT and RT + CT on cognitive function, physical performance, body composition, and BDNF levels in healthy community-dwelling older adults. Baseline data were collected after the participants became familiar with each test. The same physical trainers and researchers, with 5 years of experience, who tested and retested the participants, also followed them during the trainings. The participants were randomly allocated into the intervention groups using computer-generated random numbers.^1^ Each participant performed the tests at the same time of the day.

### Participants

The sample size was calculated using Cohen’s d (ES = 0.70) based on the power analysis of a previous study (Coelho et al., 2012). All procedures were conformed the ethical guidelines of the Declaration of Helsinki and Resolution 196/96 of the National Health Council. Participants were informed about the study procedures, risks, and benefits before giving written consent.

Participants were recruited through advertisements in regional public health centers and by direct contact. The inclusion criteria were age ≥ 60 years, living in the community, and having sufficient physical and cognitive abilities to perform the interventions. A convenience sample of 57 older adult was randomly selected using Mini-Mental State Examination (MMSE) and timed “up-and-go” cognitive test scores (Coelho-Júnior et al., 2018a). The TRT and RT + CT group was composed by 15 older adult, each one allocated by randomization.

The exclusion criteria were participants who started a physical activity program 3 months before the beginning of the study or during the study period, and individuals with a clinical diagnosis of skeletal muscle disorders and/or cardiovascular, pulmonary, neurological and/or psychiatric diseases. The MMSE score was used as criteria of exclusion (<24). In addition, the participants who were absent in more than 10% of the exercise sessions were excluded from the study.

### Procedures

#### Interventions

Interventions for RT and RT + CT were performed twice a week for 16 weeks in two phases: a 2-week familiarization period and a 14-week physical training period. In both periods, exercise sessions lasted ~60 min, including a 5-min warm-up, 50-min exercise, and a 5-min cool-down. All sessions were conducted in groups of four participants under the supervision of two experienced physical trainers. Full range-of-motion exercises were carried out at moderate intensity based on the rating of perceived exertion (RPE).

The familiarization period was established to familiarize participants with the laboratory setting, physical training, and monitoring tools. Participants executed eight resistance exercises for the major muscle groups using Nakagym equipment (São Paulo, Brazil), free weights, and body weight. Training was carried out in the morning following the order: leg press, dumbbell lateral raise, lateral pulldown, abdominal crunch, back extension, seated leg curl, bench press, and standing calf raise. Cognitive tasks were not executed during familiarization.

Both intervention groups executed the same set of exercises during the familiarization period, and the total training volume (number of sets × number of repetitions × weight lifted) was equalized between the groups. Exercise intensity and volume for leg press, seated leg curl, bench press, and lateral pulldown were increased over the training period, as follows: from week 3 to 10, two sets of 8–10 submaximal repetitions at 60% of 1-repetition maximum (1RM); from week 11 to 13, three sets of 8–10 submaximal repetitions at 60% of 1RM; on weeks 14 and 15, three sets of 8–10 submaximal repetitions at 70% of 1RM. For dumbbell lateral raise, abdominal crunch, back extension, and standing calf raise, the participants performed three sets of 12–15 submaximal repetitions at moderate-to-high intensity according to the RPE (CR-10) method (moderate = 3, high = 6; Foster et al., 2001). The targeted RPE remained the same across the training period. A 1-min rest interval between sets was adopted for all exercises.

The 1RM test was performed monthly (Brown and Weir, 2001). The test involved gradually increasing the weight lifted until 1-RM was achieved, with a maximum of five attempts and a 3-min interval between attempts. The test–retest reliability among evaluations was high (intraclass correlation coefficient of 0.99). RPE was quantified using a CR-10 scale at the end of each exercise set. If participants reported an RPE below the expected, training load was increased 2%–5% for upper extremity exercises and 5%–10% for lower extremity exercises (Illi et al., 2012). Before the tests, participants performed a 5-min warm-up on a cycle ergometer.

**Figure 1:**
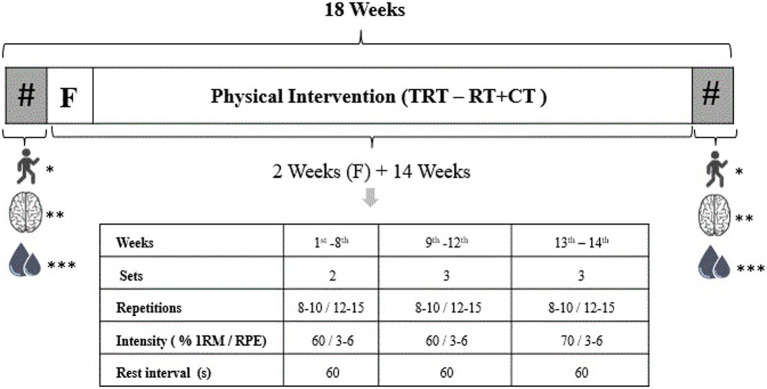
Experimental design of the study. TRT, traditional resistance training; RT + CT, resistance training combined with cognitive training; #, evaluations; F, familiarization; s, seconds; RPE, rating of perceived exertion; 1RM, 1-repetition maximum; *physical function; **cognitive function; ***body composition measurement and blood collection.

The experimental design is shown in Figure 1.

The RT + CT training involved performing resistance exercises simultaneously with a cognitive [verbal fluency (VF)] task. The VF task involved saying aloud as many words as possible of a specific category in each exercise set. Task difficulty was increased monthly by changing the categories of words, from general to specific, whereas semantic categories (e.g., animals and colors) and phonological categories (e.g., words beginning with a certain letter) were changed in each exercise set (Table 1). Participants were encouraged not to repeat words and think in new words.

**Table 1:** Examples of semantic and phonological categories used in the training program.

Sets	Repetitions									
	1st	2nd	3rd	4th	5th	6th	7th	8th	9th	10th
1st	Car	Bus	Boat	Bike	Motorcycle	Airplane	Helicopter	Train	Truck	Ambulance
2nd	Orange	Yellow	Red	Green	Black	White	Blue	Purple	Gray	Brown
3rd	United States	Australia	Canada	Germany	France	Spain	Italy	Denmark	Argentina	Brazil

Semantic categories: 1st set = Transport; 2nd set = Colors; 3rd set = Countries.

#### Assessments

Assessments were performed 1 week before (baseline) and 1 week after (post-intervention) RT protocols. During these two periods, physical performance and cognitive function were assessed on the first and second day, respectively, and body composition measurement and blood collection were performed on the third day. The participants were asked to abstain from intense physical activity during the 48 h before blood collection.

#### First Day

The physical function tests included isometric handgrip strength (IHG), 10-m walk test (10 MWT) at usual and fast pace, sit-to-stand test five times (5XSTS), timed up-and-go test (TUG), and one-leg stand. The tests were performed twice, and the highest scores were included in the analysis.

The isometric handgrip strength was measured using a hydraulic hand dynamometer grip Saehan, (SH5001) with participants sitting on a chair with shoulders adducted, elbows flexed at 90° near the trunk, and wrist in a neutral position. The contralateral arm remained beside the trunk. The participants were asked to squeeze the handgrip as hard as they could for 4–6 s using the dominant hand (Mathiowetz et al., 1984). Relative IHG was calculated by dividing IHG by the BMI.

The 10-m walk test involved walking 12 m at usual and fast pace. Before the test, both feet remained on the starting line. Measurement was started when one foot reached the 1-m line and was stopped when one foot reached the 11-m line. The 1-m intervals at the beginning and end of the course were used for acceleration and deceleration, respectively, and were not included in the analysis (Sampaio et al., 2013).

The sit-to-stand test required rising from an armless chair (total height, 87 cm; seat height, 45 cm; width, 33 cm) five times as fast as possible with arms crossed in front of the body. The stopwatch (1/100 s accuracy) was started when participants rose from the chair and was stopped when they sat on the chair after the fifth repetition (Sampaio et al., 2013).

For the timed up and go test participants were instructed to get up and walk as fast as possible along a 3-m line demarcated on the floor upon hearing the word “go” without compromising their safety, turn around, return to the original position, and sit down. Timing was started when participants rose from the chair and was stopped when their backs touched the backrest of the chair (Coelho-Júnior et al., 2018b).

In the one-leg stand test, one leg remained on the floor while the other was raised with the knee flexed at 90° and arms crossed in front of the chest. The stopwatch was activated when each participant lifted one foot and was stopped when the foot touched the floor again. The maximum time allowed to perform the test on each leg was 30 s (Briggs et al., 1989).

#### Second Day

Cognitive function was assessed based on VF (Troyer et al., 1997), dual task [TUG + cognitive test (TUG-cog)], (Coelho-Júnior et al., 2018b), short-term memory [Scenery Picture Memory Test (SPMT)], (Takechi and Dodge, 2010), executive function, visual attention, and task switching [Trail-Making Test (TMT)] (Tombaugh, 2004).

The Verbal Fluency was assessed using semantic and phonological tests. Participants were requested to say aloud the name of as many animals (semantic domain) and words that began with the letter “A” (phonological domain) as possible for 2 min (1 min for each domain).

In the TUG-cog, participants were asked to say the names of animals (Coelho-Júnior et al., 2018a).

The Scenery Picture Memory Test involved analyzing an image of a living room with 23 objects for 1 min, memorizing as many items as possible, and recalling them after a 1 min interval. There was no time limit to complete the test (Takechi and Dodge, 2010).

The Trail-Making Test is divided into two parts, TMT-A and TMT-B. TMT-A consisted of drawing a line connecting a sequential set of numbers (1–25), whereas TMT-B consisted of drawing a line connecting sequential numbers (1–13) and letters (A-L) and alternating numbers and letters (e.g., 1a, 2b, 3c, 4d). Participants were required to perform the test as quickly as possible (Tombaugh, 2004).

#### Third Day

Body composition was measured, and fasting blood samples were collected. Participants were asked to avoid physical activity for at least 48 h before blood collection.

Body composition (total body mass, lean body mass, appendicular skeletal muscle mass, and fat mass) was estimated using an electrical bioimpedance data acquisition system (Tanita BC-108, Tokyo, Japan). This system uses an electric current at a frequency of 50 kHz to directly measure the amount of intracellular and extracellular water in the body. The device has eight electrodes, of which four are placed on the soles of the feet and four are placed on the palm (Yamada et al., 2013).

Blood samples were collected by venous puncture in vacutainer tubes (BD Bioscience, Franklin Lakes, New Jersey) containing ethylenediaminetetraacetic acid and were centrifuged at 3,000 rpm at 4°C for 10 min. Plasma was aliquoted and stored at −80°C until analysis. Plasma BDNF was measured using an ELISA kit (Sigma-Aldrich, Poole, United Kingdom; Ref: RAB0026) following the manufacturer’s instructions. All tests were performed in duplicate.

### Statistical Analysis

Data are shown as mean ± standard deviation, except for sex, which are shown as *n* (%). The normality of the distributions was assessed using the Shapiro–Wilk test. All data were normally distributed. Baseline values between groups were compared using Student’s *t*-test. Differences between groups and periods were assessed *via* two-way (group × time) ANOVA. Tukey post-hoc tests were carried out when necessary. The relationship between cognitive functions and BDNF levels was evaluated using Pearson correlation. The effect size of the results was calculated using Cohen’s d. The level of significance was set at 5% (*p* < 0.05). All analyses were performed using STATISTICA version 6.0 (StatSoft Inc).

## Results

**Figure 2:**
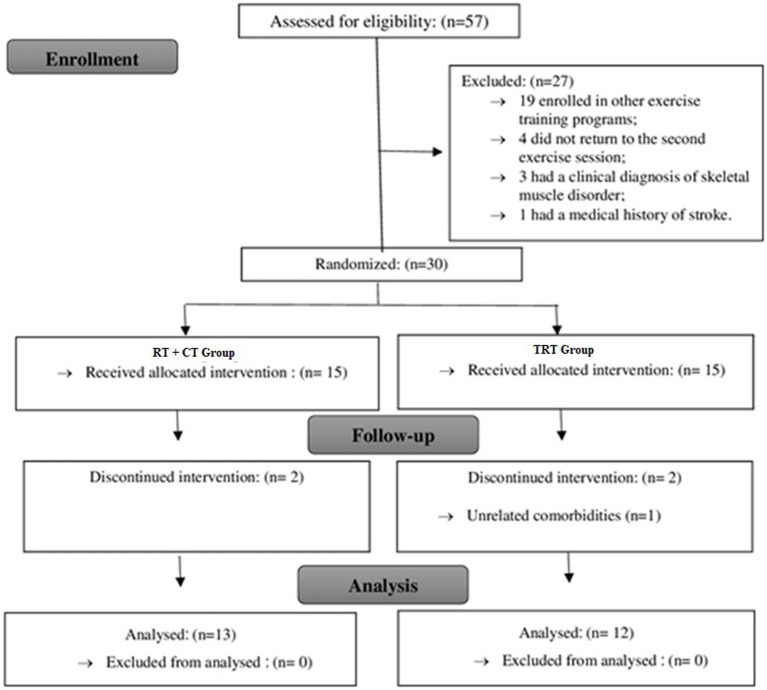
Flowchart of the present study. TRT, traditional resistance training; RT + CT, resistance training combined with cognitive training.

Fifty-seven older adults were recruited. Twenty-seven participants were excluded according to the eligibility criteria, and 30 older adults were randomly assigned to the TRT and RT + CT groups. Five participants were excluded from the analysis because of non-adherence to the program (four cases) and the initiation of a dermatological treatment (one case). Hence, 25 participants completed the training program and were included in the final analysis (Figure 2).

The characteristics of the study participants are shown in Table 2. There were no significant differences between the groups at baseline.

**Table 2:** General characteristics of the study groups at baseline.

Variable	RT + CT (*n* = 13)	TRT (*n* = 12)	*p*-value
Age (years)	66.3 ± 4.6	70.0 ± 8.1	0.368
Formal education (years)	16.3 ± 2.0	14.8 ± 4.0	0.312
Men (*n*, %)	4 (31)	3 (25)	0.748
Height (cm)	162.0 ± 0.0	162.0 ± 0.0	0.986
Body weight (kg)	74.1 ± 14.0	72.2 ± 12.0	0.801
BMI (kg/m^2^)	28.0 ± 3.7	27.4 ± 3.8	0.693
MMSE (score)	28.1 ± 1.0	28.4 ± 1.3	0.861
TUG cognitive test (s)	7.9 ± 1.9	7.6 ± 0.9	0.548

BMI, body mass index; RT + CT, resistance training combined with cognitive training; MMSE, Mini-Mental State Examination; TRT, traditional resistance training; TUG, timed up-and-go test.

The effects of TRT and RT + CT on body composition and physical function are shown in Table 3. A time effect was observed and the post-hoc showed improvement in both groups. TRT and RT + CT improved absolute and relative IHG, 10MWT at a fast pace, 5XSTS, and TUG scores and increased total training volume and maximum muscle strength on the leg press, lateral pulldown, and leg curl (all, *p* < 0.05). RT in the presence or absence of CT had no significant effect on body composition. Moreover, there were no significant differences in physical function scores between the two groups.

**Table 3:** Effects of TRT and RT + CT on body composition and physical function.

Variables	RT + CT (*n* = 13)				TRT (*n* = 12)				Time	Group × time
	Pre	Post	ES	Δ (%)	Pre	Post	ES	Δ (%)		
*Physical function*										
Usual WS (m/s)	1.3 ± 0.1	1.4 ± 0.1	0.1	7.7	1.3 ± 0.2	1.4 ± 0.2	0.5	7.7	0.06	0.97
Fast WS (m/s)	1.7 ± 0.1	1.9 ± 0.2^*^	1.3	11.7	1.6 ± 0.3	1.9 ± 0.2^*^	1.2	18.7	0.001	0.29
5XSTS (s)	9.5 ± 2.5	7.3 ± 1.5^*^	1.1	23.1	10.1 ± 1.5	7.6 ± 1^*^	2.0	24.7	0.001	0.75
TUG (s)	6.6 ± 0.8	5.5 ± 0.8^*^	1.4	16.6	7.0 ± 1.2	6.1 ± 0.6^*^	1.0	12.8	0.001	0.19
Absolute IHG (kg)	26.8 ± 7.6	31.5 ± 9.1^*^	0.5	17.5	19.8 ± 3.7	26.6 ± 4.9^*^	1.5	34.4	0.001	0.07
Relative IHG (kg/BMI)	0.9 ± 0.2	1.1 ± 0.3^*^	0.8	22.2	0.7 ± 0.1	0.9 ± 0.2^*^	1.3	28.5	0.001	0.09
1-RM leg press (kg)	164 ± 46	246 ± 56^*^	1.6	50	151 ± 39	235 ± 60^*^	1.7	55.6	0.001	0.73
1-RM lateral pulldown (kg)	32.6 ± 11.9	43.0 ± 10.9^*^	0.9	31.9	28.6 ± 4.8	36.5 ± 4.5^*^	1.7	27.6	0.001	0.59
1-RM leg curl (kg)	30.7 ± 8.3	40.0 ± 11.5^*^	0.9	30.2	27.6 ± 8.7	38.9 ± 9.9^*^	1.2	40.9	0.001	0.15
1-RM bench press (kg)	26.4 ± 6.9	27.3 ± 4.6	0.1	3.4	31.6 ± 12.8	34.1 ± 8.4	0.2	7.9	0.49	0.83
Total volume^#^	3,346 ± 869	8,135 ± 1,858^*^	3.5	143.1	3,091 ± 748	7,637 ± 1,648^*^	3.7	147	0.001	0.47
*Body composition*										
Body weight (kg)	73.9 ± 12.3	73 ± 12.0	0.07	0.9	72.4 ± 13.0	71.4 ± 12.4	0.1	1.4	0.03	0.86
BMI (kg/m^2^)	28.1 ± 3.8	27.9 ± 3.6	0.05	0.5	27.5 ± 3.7	27.1 ± 3.6	0.1	1.3	0.10	0.57
LBM (kg)	46.3 ± 10.1	45.8 ± 9.3	0.06	0.8	44.8 ± 10.1	44.1 ± 9.3	0.1	1.2	0.09	0.88
ASMM (kg)	20.9 ± 6.1	20.1 ± 5.7	0.13	3.5	19.0 ± 4.4	19.0 ± 4.5	0.0	0.2	0.72	0.14
Fat mass (%)	38.0 ± 7.0	37.9 ± 6.3	0.03	0.9	36.7 ± 7.6	36.4 ± 8.1	0.0	1.0	0.87	0.92

1RM, 1-repetition maximum, 5XSTS, five-times sit-to-stand test, ASMM, appendicular skeletal muscle mass, BMI, body mass index; LBM, lean body mass; RT + CT, resistance training combined with cognitive training; ES, effect size; IHG, isometric handgrip strength; TRT, traditional resistance training; TUG, timed up-and-go test; WS, walking speed.

* Main effect of time (Post-hoc), *p* < 0.05.

# Sets × repetitions × weight.

**Figure 3:**
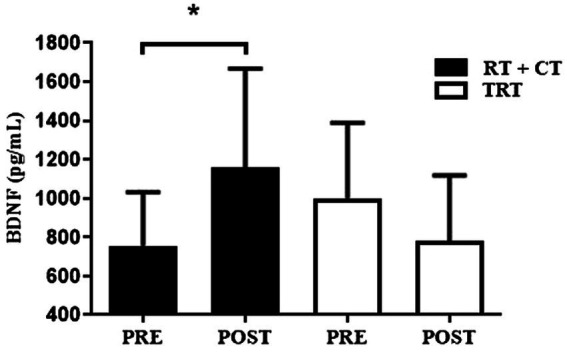
Effects of resistance training on plasma BDNF levels. BDNF, brain-derived neurotrophic factor; TRT, traditional resistance training; RT + CT, resistance training combined with cognitive training. *Difference between pre- and post-training (post-hoc test), *p* < 0.05.

The effects of TRT and RT + CT on cognitive functions are shown in Table 4. A time effect was observed for TUG-cog and the post-hoc identified significant difference for both groups, without significant differences between the groups. A time effect was found for semantic and phonological VF and SPMT, the post-hoc test identified that only RT + CT improved these variables (all *p* < 0.05). The BDNF level showed a significant group x time effect and the post-hoc showed that RT + CT (Pre-training 757 ± 260, Post-Training 1,158 ± 483) presented increased BDNF level at the end of the intervention compared to TRT (Pre-training 1,001 ± 367, Post-Training 780 ± 320), the difference can be seen in the Figure 3.

**Table 4:** Effects of TRT and RT + CT on cognitive functions.

Variables	RT + CT (*n* = 13)				TRT (*n* = 12)				Time	Group × Time
	Pre	Post	ES	Δ%	Pre	Post	ES	Δ%		
*Cognitive functions*										
SVF (words/min)	17.5 ± 5.6	22.4 ± 5.1^*^	0.9	28.0	18.5 ± 4.9	20.0 ± 5.4	0.3	0.8	<0.001	0.05
PVF (words/min)	13.3 ± 5.3	17.5 ± 6.3^*^	0.7	31.5	14.5 ± 5.1	15.7 ± 5.6	0.2	8.2	0.09	0.05
TUG-cog (s)	7.6 ± 0.9	6.2 ± 0.5^*^	1.3	18.4	7.9 ± 1.6	6.7 ± 0.9^*^	0.9	15.1	<0.001	0.56
TMT-A (s)	47.4 ± 16.8	42.0 ± 14.8	0.3	11.3	39.1 ± 8.6	39.0 ± 17.2	0.0	0.3	0.06	0.67
TMT-B (s)	87.1 ± 44.4	85.0 ± 32.4	0.0	2.4	103.9 ± 50.4	124.9 ± 71.3	0.3	20.2	0.52	0.52
SPMT (points)	15.6 ± 3.8	17.1 ± 3.6^*^	0.4	9.6	15.4 ± 3.7	16.4 ± 4.1	0.3	0.6	0.03	0.67
BDNF (pg/mL)	757 ± 260	1,158 ± 483^*^	1.0	52.9	1,001 ± 367	780 ± 320	0.6	22	0.41	0.001

BDNF, brain-derived neurotrophic factor; RT + CT, resistance training combined with cognitive training; ES, effect size; SPMT, Scenery Picture Memory Test; TMT-A, Trail Making Test A; TMT-B, Trail Making Test B; TRT, traditional resistance training; SVF, semantic verbal fluency; PVF, phonological verbal fluency.

* Main effect of time (Post-hoc), *p* < 0.05.

**Figure 4:**
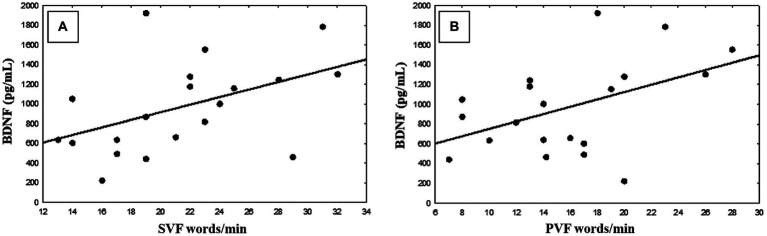
Pearson correlation between brain-derived neurotrophic factor (BDNF) and semantic verbal fluency (SVF) scores **(A)** and phonological verbal fluency (PVF) scores **(B)**.

The relationship between peripheral BDNF levels and cognitive scores after training was evaluated using Pearson correlation (Figure 4). There was a positive significant correlation between BDNF levels with SVF (*r* = 0.463; *p* = 0.039, *n* = 25) (A) and PVF (*r* = 0.461; *p* = 0.040, *n* = 25). No other significant associations were observed.

## Discussion

Our findings indicated that RT in the presence or absence of CT improved physical function in healthy older adults. However, RT + CT but not TRT improved cognition, semantic and phonological VF, and plasma BDNF levels. These results indicate that RT + CT improves cognitive function in physically healthy older adults and that BDNF may mediate this effect.

Both TRT and RT + CT improved physical performance and maximum muscle strength on resistance exercises. Notably, 1RM leg-press, IHG, 5XSTS, 10MWT, and TUG scores increased by approximately 53%, 25%, 24%, 15%, and 15%, respectively, consistent with a previous study (Coelho-Júnior et al., 2018b) and a meta-analysis (Guizelini et al., 2018), wherein RT improved physical function in community-dwelling older adults. Hence, our findings confirm that RT enhances physical function in older adults.

These results may have direct implications on older adults’ quality of life, given that reduced physical performance predisposes to the development of disabilities, frailty (Hoogendijk et al., 2019), and sarcopenia (Cruz-Jentoft et al., 2019), which are associated with increased rates of falls and fractures, depressive symptoms, cognitive decline, hospitalization, nursing home placement, and death (Fitzpatrick et al., 2007).

There is controversy regarding the effects of RT on muscle mass in older adults (Borde et al., 2015; Prestes et al., 2015; Coelho-Júnior et al., 2019), most likely because these effects are protocol-dependent (Borde et al., 2015) and the best protocol is still unknown. Nonetheless, evidence suggests that high-intensity RT (Kalapotharakos et al., 2004) and RT until concentric muscle failure (Jenkins et al., 2016) are more likely to promote muscle hypertrophy in older adults, which could explain our results, since we apply moderate/high intensity loads training.

The present findings indicate that RT + CT improved semantic and phonological VF by 28% and 31%, respectively, and TRT and RT + CT increased TUG-cog scores by 18% and 15%, respectively.

There is also controversy regarding the effects of RT on cognitive function in older adults. Our results are supported by Tsutsumi et al. (1997) and Kimura et al. (2010), wherein RT did not significantly improve neurocognitive function in physically healthy older adults. In contrast, Cassilhas et al. (2007) reported that moderate-intensity RT (50% 1RM) and high-intensity RT (80% 1RM) improved memory performance and verbal concept formation in older adults. In addition, Liu-Ambrose et al. (2010) found that RT performed once or twice a week improved executive functions (selective attention and conflict resolution) in community-dwelling older women, and Coelho-Júnior et al. (2020) observed that TRT in the presence or absence of power training improved cognitive function, short-term memory, and dual-task performance in older women.

A possible explanation for these contradictory results is the difference in the duration of intervention, given that the latter three studies evaluated RT performance for at least 22 weeks, whereas the former two studies and the present investigation assessed RT performance for up to 16 weeks. In this respect, interventions of at least 6 months seem to be necessary to elicit significant changes in cognitive parameters (Vellas et al., 2007).

Another explanation may be related to the near-transfer effect in cognitive training. Near transfer of training is defined as a situation where the stimulus for the original learning event is similar to that for the transfer event (i.e., task; Royer, 1979; Sala et al., 2019). In the present study some part of cognitive outcomes showed improvement of verbal fluency, corroborating the near-transfer effect training theory, specific adaptations by similar stimulus (e.g., verbal fluency improved by verbal fluency). And the opposite, far transfer effect is defined as a situation where the stimulus for the original learning event is distinct to that for the transfer event (Royer, 1979), which it is rare to occur (Sala et al., 2019). Ours results endorsed it, with the improvement of VF but not memory (short memory test) and attention in the RT + CT group. Therefore, the present study corroborates the difficulty in generating far transfer for other cognitive adaptation that are not similar task and/or type of stimulus (Sala et al., 2019).

The better result (shorter time test) for TUG-cog scores may be due to enhanced motor performance, rather than better cognitive functioning, since both TRT and RT + CT improved TUG-cog scores (*ES* of 0.9 and 1.3, respectively).

Neurophysiological mechanisms underlying improvements in physical and cognitive performance have been proposed, including the “guided plasticity facilitation” framework, which postulates that the sequential or simultaneous execution of physical and cognitive tasks has synergistic benefits (Herold et al., 2018). This framework emerges from “facilitation effect” by physical exercises and the “guidance effect” by cognitive exercises, neural activation (e.g., increasing in neural growth factors, e.g., BDNF) and mechanisms of new synapses, respectively. Therefore, “guided plasticity facilitation” is important to induce neuroplasticity changes (Herold et al., 2018). BDNF may mediate morpho functional changes induced by CT in brain regions involved in cognition (Håkansson et al., 2017) which might explain our findings that the increased of BDNF levels may mediate the improvement in cognitive tests after 16-week in the RT + CT participants. Notably, the null effect of traditional resistance training on BDNF levels is supported by other studies (Fragala et al., 2014; Prestes et al., 2015), allowing us to hypothesize that resistance training combined with cognitive training (simultaneously) induces an increase in BDNF levels. In this respect, Damirchi et al. (2018) measured rest blood BDNF levels after 8 weeks of aerobic physical training; cognitive training; and dual-task training (aerobic + cognitive training). They showed that the latter two interventions (cognitive and Dual-Task) increased BDNF levels, consistent with our results.

Our study has the following limitations: (1) treatment effects may have been overestimated or underestimated because of the lack of a control and a CT group; (2) the correlation between BDNF levels and cognitive improvement may be mediated by physiological mechanisms; however, this study did not establish cause-effect relationships.

In conclusion, the results showed that a 16-week moderate-intensity RT program alone or combined with CT improved physical performance in healthy older adults. However, only RT + CT improved cognitive function and increased plasma BDNF levels. In addition, this kind of CT showed a possible association “near-transfer effect” with SVF and PVF test, but no “far transfer-effect.”

## Data availability statement

The raw data supporting the conclusions of this article will be made available by the authors, without undue reservation.

## Ethics statement

The studies involving human participants were reviewed and approved by the Institutional Human Research Ethics Committee of the UNICAMP under the protocol number 2.524.553. The patients/participants provided their written informed consent to participate in this study.

## Author contributions

LC and MU: conceptualization. LC, VL, JB, and AG: data collection. LC, VL, JB, HC-J, and MU: formal analysis. LC, VL, EL, and MU: funding acquisition. LC, VL, JB, and EL: investigation and methodology. LC, VL, and MU: project administration. CT and MU: supervision. LC, MU, and HC-J: writing–original draft. LC, HA, MU, HC-J, and CT: writing–review and editing. All authors contributed to the article and approved the submitted version.

## Funding

This work was supported by the National Council for Scientific and Technological Development – Brazil (CNPq) (#131240/2018-8).

## Conflict of Interest

The authors declare that the research was conducted in the absence of any commercial or financial relationships that could be construed as a potential conflict of interest.

## Publisher’s Note

All claims expressed in this article are solely those of the authors and do not necessarily represent those of their affiliated organizations, or those of the publisher, the editors and the reviewers. Any product that may be evaluated in this article, or claim that may be made by its manufacturer, is not guaranteed or endorsed by the publisher.

## Footnotes

^1^ http://www.randomization.com
